# Effects of Organic Amendments Combined with Mineral Fertilizer on Soil Properties and Crop Yield in a Maize–Soybean Rotation System on Meadow Albic Soil

**DOI:** 10.3390/plants15091412

**Published:** 2026-05-06

**Authors:** Yubo Sun, Qu Chen, Hao Li, Yuzhe Wu, Da Song, Lining Dou, Meng Hou, Shoukun Song, Jingru Zheng, Yuxian Zhang, Mingcong Zhang, Tangzhe Nie, Xingchao Liu, Mengxue Wang

**Affiliations:** 1 College of Agronomy, Heilongjiang Bayi Agricultural University, Daqing 163000, China; 15776586250@163.com (Y.S.); 17604530392@163.com (H.L.); 18545559306@163.com (Y.W.); songda200205@163.com (D.S.); 13836216629@163.com (L.D.); houmeng@byau.edu.cn (M.H.); a13199436890@163.com (S.S.); lvsebaowen513@163.com (J.Z.); zhangmingcong@163.com (M.Z.); 2 Engineering Research Center of Agricultural Microbiology Technology, Ministry of Education & Heilongjiang Provincial Key Laboratory of Ecological Restoration and Resource Utilization for Cold Region & School of Life Sciences, Heilongjiang University, Harbin 150080, China; 18345743002@163.com; 3 National Multigrain Engineering and Technology Center, Daqing 163000, China; 4 School of Water Conservancy and Electric Power, Heilongjiang University, Harbin 150080, China; 2019036@hlju.edu.cn; 5 School of Water Conservancy and Civil Engineering, Northeast Agricultural University, Harbin 150030, China; dnlxc@neau.edu.cn

**Keywords:** Albic Luvisols, nutrient availability, aggregate fractions, phosphatase activity, alkali-hydrolyzable nitrogen, rotation cropping

## Abstract

Meadow albic soils in the Sanjiang Plain of Northeast China are characterized by a compact plow layer, weak structural stability, low organic matter content, and limited nutrient availability, which restrict crop productivity in maize–soybean rotation systems. A two-year field experiment (2023–2024) was conducted to compare the effects of mineral fertilizer alone (CF) and CF combined with carbon-based organic fertilizer (CF+COF), humic acid organic fertilizer (CF+HA), or biochar-based fertilizer (CF+BC) on soil properties and crop yield. Soil aggregate composition, pH, organic carbon, total nitrogen, alkali-hydrolyzable nitrogen, available phosphorus, available potassium, and enzyme activities were measured together with yield and 100-grain weight. Compared with CF alone, the combined application of organic amendments generally improved soil properties and increased crop yield, although the magnitude and pattern of response differed among materials. CF+COF was more effective in increasing the proportion of medium-sized aggregates, enhancing alkali-hydrolyzable nitrogen and some enzyme activities, and achieving relatively high yields in both maize and soybean seasons. CF+HA showed comparatively balanced effects on aggregate composition and nutrient availability, whereas CF+BC was more effective in maintaining relatively high soil pH, increasing available phosphorus, and promoting larger aggregates at later growth stages. Overall, all three organic amendments combined with mineral fertilizer were beneficial for improving meadow albic soil and increasing crop yield, with CF+COF showing the best overall performance under the conditions of this study.

## 1. Introduction

Northeast China is one of the major commercial grain-producing regions of China, and the quality of its arable land and the long-term stability of its soil resources are critical to sustainable crop production. Maize–soybean rotation is a dominant cropping system in this region and plays an important role in maintaining soil fertility and stabilizing crop yields [1,2]. However, the widely distributed meadow albic soils in this area, classified as Albic Luvisols, are characterized by a compact and crust-prone plow layer, low organic matter content, weak nutrient buffering capacity, and poor nutrient availability, all of which impose major constraints on crop productivity in rotation systems [3]. Specifically, plow-pan compaction restricts soil aeration and water infiltration, while low organic matter content further weakens the soil’s capacity to retain water and nutrients. In addition, poor nutrient availability limits the continuous uptake and utilization of nutrients by crops [4,5]. Under long-term intensive cultivation, reliance on mineral fertilizers alone is often insufficient to sustainably improve the structure and fertility of meadow albic soils and may even accelerate soil degradation and reduce nutrient use efficiency, thereby limiting yield potential in rotation systems [6,7]. Therefore, developing soil fertility management strategies that simultaneously address the major constraints of meadow albic soils while coordinating soil improvement with yield enhancement is essential [8,9].

Crop yields in rotation systems are not only affected by fertilization level, but are also closely related to the structural condition of the plow layer, nutrient availability, and soil enzyme activity [10,11]. Meadow albic soils have weak aggregate stability and are prone to structural breakdown under rainfall, irrigation, and tillage disturbances, which in turn affects soil aeration, water infiltration, and nutrient retention and supply [12]. When soil structure deteriorates or nutrient availability is insufficient, high fertilizer inputs may still fail to translate into effective nutrient uptake and use by crops, thus limiting yield formation [13]. In addition, soil enzyme activity is an important indicator reflecting soil biochemical activity and nutrient cycling processes [14]. Sucrase, urease, and phosphatase participate in soil C, N, and P transformations, respectively, while catalase is closely related to redox-related biochemical processes in soils; thus, changes in soil enzyme activity can provide biochemical evidence for crop yield formation [15]. Maize is particularly sensitive to a continuous nutrient supply from tasseling to grain filling [16]. Soybean, although capable of biological N fixation, still relies heavily on the adequate availability of P, K, and secondary/micronutrients from flowering-pod setting to seed filling; insufficient supply in this period restricts pod formation and grain filling [17]. Therefore, in maize–soybean rotation systems, optimizing soil structure and nutrient status is critical for achieving yield increases [18].

The application of organic amendments is widely regarded as an effective strategy for alleviating soil constraints, improving soil properties, and enhancing crop productivity [19]. However, different organic amendments vary substantially in their composition and modes of action. In this study, COF refers to a commercial carbon-based organic fertilizer product, whereas BC refers to a biochar-based fertilizer applied as a distinct amendment treatment. Although both materials contain carbon-rich components, they differ in product type and nutrient characteristics. COF generally contains relatively labile organic fractions and available nutrients, which may increase soil organic matter content and nutrient supply and promote aggregate formation in the short term [20,21]. Humic acid (HA), owing to its strong surface activity and complexation capacity, can regulate soil colloidal properties and nutrient availability and may also improve aggregate stability as well as soil water- and nutrient-holding capacity [22,23]. Biochar (BC), because of its stable carbon structure and porous properties, has potential advantages in regulating soil pH, adsorbing and gradually releasing nutrients, and improving soil water-holding capacity [24]. Although the beneficial effects of organic amendments on soil improvement have been widely reported [25], important knowledge gaps remain for meadow albic soils under maize–soybean rotation in Northeast China. First, systematic comparisons among carbon-based organic fertilizer (COF), humic acid organic fertilizer (HA), and biochar-based fertilizer (BC) under the same field conditions are still limited. Second, few studies have jointly evaluated soil aggregate characteristics, chemical properties, enzyme activities, and crop yield responses, making it difficult to clarify the differential effects of these amendment products on soil improvement and yield formation [26]. In addition, many previous studies have focused on a single amendment or a single crop season, which limits understanding of how these materials influence soil constraints and crop productivity under rotation systems. Therefore, fixed-site field experiments are needed to compare the effects of different organic amendments on meadow albic soil properties and crop yield in maize–soybean rotations.

Based on this, a two-year field experiment was conducted from 2023 to 2024 in a typical meadow albic soil area of the Sanjiang Plain, Heilongjiang Province. Five treatments were established: no fertilizer (CK), conventional mineral fertilizer (CF), and CF combined with carbon-based organic fertilizer (CF+COF), humic acid organic fertilizer (CF+HA), or biochar-based fertilizer (CF+BC). At key growth stages of maize and soybean, soil aggregate composition, pH, organic carbon, alkali-hydrolyzable nitrogen, available phosphorus, and available potassium were measured together with the activities of sucrase, catalase, urease, and phosphatase, and these were analyzed in relation to crop yield and 100-grain weight. We hypothesized that different organic amendments combined with mineral fertilizer would differentially affect soil aggregate composition, nutrient availability, enzyme activities, and crop yield in the maize–soybean rotation system on meadow albic soil. The objectives of this study were to: (1) compare the differential effects of the three organic amendments on soil structural improvement and nutrient availability in meadow albic soil and (2) clarify how the combined application of mineral fertilizer with different organic amendments affects soil physicochemical properties, enzyme activities, and their relationships with maize and soybean yield formation.

## 2. Results

### 2.1. Effects of Different Fertilization Treatments on Soil Aggregate Structure

**Figure 1:**
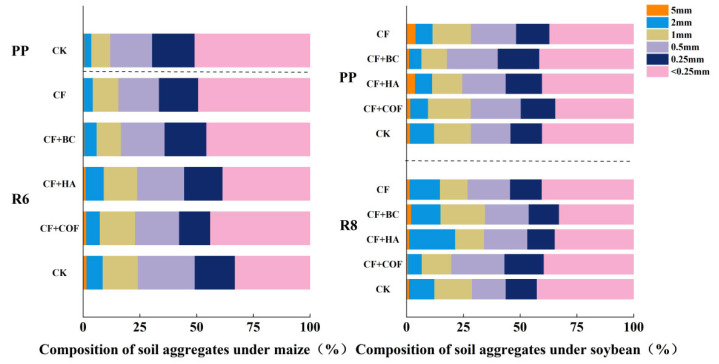
Effects of combined application of different organic materials and mineral fertilizer on soil aggregate structure of meadow albic soil. Note: Treatments: CK, no fertilizer; CF, mineral fertilizer; CF+COF, mineral fertilizer + carbon-based organic fertilizer; CF+HA, mineral fertilizer + humic acid organic fertilizer; CF+BC, mineral fertilizer + biochar-based fertilizer.

As shown in Figure 1, overall, the CF+COF treatment was more conducive to increasing the proportion of 0.5–1 mm aggregates; CF+BC produced the greatest increase in >2 mm macroaggregates at the R8 stage; and CF+HA most prominently enhanced the proportion of 1–2 mm aggregates at R8.

At the R6 stage of the maize season, the CK treatment had the highest proportion of >2 mm aggregates. Under CF+COF, the proportion of 1–2 mm aggregates decreased by 5.4% compared with CK, while the proportion of 0.5–1 mm aggregates decreased by 17.1%, and that of >2 mm aggregates increased by 12.9%. Under CF+HA and CF+BC, the proportions of >2 mm aggregates were both lower than that of CK, decreasing by 23.3% and 46.5%, respectively; among them, CF+BC had the lowest proportion of 1–2 mm aggregates, 27.5% lower than CK. The CF treatment showed the highest proportion of <0.25 mm microaggregates, 53.5% higher than CK, indicating limited structural improvement.

At the R8 stage of the soybean season, differences among treatments were further reflected in changes in the proportions of macroaggregates and microaggregates. The >2 mm aggregate proportion under CF+BC was 15.7% higher than CK, the highest among all treatments. CF+HA showed the highest proportion of 1–2 mm aggregates, 18.2% higher than CK, while its proportion of <0.25 mm microaggregates was 42.8% lower than CK, indicating marked structural improvement. CF+COF increased the proportion of 0.5–1 mm aggregates by 58.0% relative to CK, suggesting a promoting effect on medium-sized aggregates. In contrast, CK and CF still maintained relatively high proportions of <0.25 mm microaggregates, with limited improvement in aggregate structure.

Within the maize season, treatment differences were mainly reflected in the 0.5–2 mm fraction, whereas within the soybean season, CF+HA and CF+BC were more frequently associated with higher proportions of aggregates >1 mm. At the soybean R8 stage, CF+HA increased the proportion of 1–2 mm aggregates and reduced the proportion of microaggregates, whereas CF+BC showed the highest proportion of the >2 mm fraction among the fertilized treatments.

### 2.2. Effects of Different Fertilization Treatments on Soil Chemical Properties

#### 2.2.1. Effects of Different Fertilization Treatments on Soil pH

**Figure 2:**
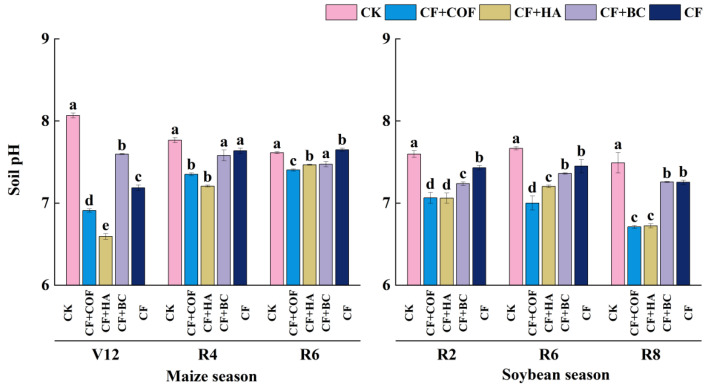
Effects of combined application of different organic materials and mineral fertilizer on pH of meadow albic soil. Note: Identical letters within the same group indicate no statistically significant difference (*p* > 0.05), while different letters indicate significant differences (*p* < 0.05). Treatments: CK, no fertilizer; CF, mineral fertilizer; CF+COF, mineral fertilizer + carbon-based organic fertilizer; CF+HA, mineral fertilizer + humic acid organic fertilizer; CF+BC, mineral fertilizer + biochar-based fertilizer.

Figure 2 shows that soil pH varied considerably among treatments across crop stages. Overall, CK maintained relatively higher pH values, whereas the combined application of organic amendments with mineral fertilizer generally reduced pH to different extents. Among the fertilized treatments, CF+BC tended to maintain a relatively higher pH, especially at the later soybean stage.

At the V12 stage of the maize season, the pH under CK was the highest and significantly greater than that of the other treatments. The pH under CF+BC ranked second, 5.8% lower than CK. The CF treatment reduced pH by 10.9% relative to CK, while CF+COF and CF+HA showed the lowest pH values, 14.3% and 18.3% lower than CK, respectively. At the R4 and R6 stages, CK still maintained relatively high pH values, whereas CF+HA showed lower pH, decreasing by 7.2% and 1.9% compared with CK, respectively. The pH under CF+COF decreased by 5.4% and 2.8% relative to CK at these two stages.

During the soybean season, soil pH generally showed a downward trend. At the R2 stage, CK again had the highest pH; CF+BC was second, 4.7% lower than CK, while CF+COF and CF+HA reduced pH by 7.0% and 7.1%, respectively. At the R6 stage, CK still showed the highest pH; CF+BC was 4.0% lower than CK, and CF+COF had the lowest pH, 8.7% lower than CK. At the R8 stage, CK exhibited the highest pH; CF+BC remained the second-highest, 3.1% lower than CK, whereas CF+COF and CF+HA decreased pH by 10.4% and 10.2% compared with CK, respectively.

#### 2.2.2. Effects of Different Fertilization Treatments on Alkali-Hydrolyzable Nitrogen

**Figure 3:**
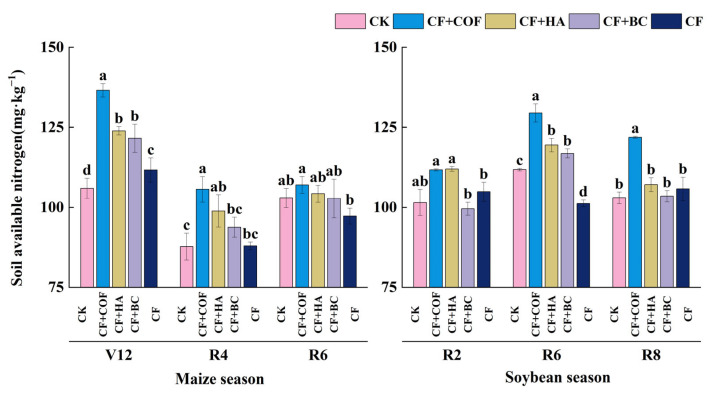
Effects of combined application of different organic materials and mineral fertilizer on alkali-hydrolyzable nitrogen content in meadow albic soil. Note: Identical letters within the same group indicate no statistically significant difference (*p* > 0.05), while different letters indicate significant differences (*p* < 0.05). Treatments: CK, no fertilizer; CF, mineral fertilizer; CF+COF, mineral fertilizer + carbon-based organic fertilizer; CF+HA, mineral fertilizer + humic acid organic fertilizer; CF+BC, mineral fertilizer + biochar-based fertilizer.

Treatment effects on alkali-hydrolyzable N varied markedly with growth stage (Figure 3). Among the amendment treatments, CF+COF generally showed the strongest enhancement during most soybean growth stages, whereas CF+HA had a more pronounced effect in the early maize season. In contrast, CF+BC showed relatively small fluctuations across stages.

At the V12 and R4 stages of the maize season, CF+COF had the highest alkali-hydrolyzable N content, 28.9% and 20.4% higher than CK, respectively. CF+HA ranked second, with increases of 17.0% and 12.7% over CK; CF+BC increased alkali-hydrolyzable N by about 14.7% and 6.9% relative to CK. By the R6 stage, differences among treatments narrowed; CF+COF remained the highest, about 3.9% above CK, while CF was the lowest, about 5.5% lower than CK. At maturity, treatment differences further decreased, but CF+COF still maintained a relatively high level throughout the maize season.

In the soybean season, treatment differences in alkali-hydrolyzable N were more pronounced at the mid–late growth stages. At R2, CF+HA showed the highest value, 10.3% higher than CK; CF+COF was slightly lower, 10.0% higher than CK; CF increased alkali-hydrolyzable N by 3.3%, while CF+BC was the lowest, 1.9% lower than CK. At R6, CF+COF peaked, 15.8% higher than CK; CF+HA was 6.9% higher than CK; CF was the lowest, 9.4% lower than CK. At R8, CF+COF still led all treatments, with an 18.4% increase over CK.

#### 2.2.3. Effects of Different Fertilization Treatments on Available Phosphorus

**Figure 4:**
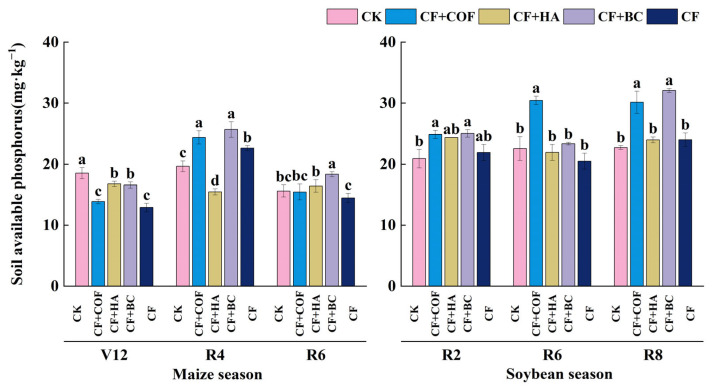
Effects of combined application of different organic materials and mineral fertilizer on available phosphorus content in meadow albic soil. Note: Identical letters within the same group indicate no statistically significant difference (*p* > 0.05), while different letters indicate significant differences (*p* < 0.05). Treatments: CK, no fertilizer; CF, mineral fertilizer; CF+COF, mineral fertilizer + carbon-based organic fertilizer; CF+HA, mineral fertilizer + humic acid organic fertilizer; CF+BC, mineral fertilizer + biochar-based fertilizer.

Available phosphorus responded differently among treatments and sampling stages (Figure 4). In general, CF+BC showed the strongest increase at the R8 stage of the soybean season, whereas CF+COF produced more pronounced enhancement at the R2 and R6 stages. CF+HA also increased available P to some extent at R6.

At the V12 stage of the maize season, the CK treatment had the highest available P content. CF+HA and CF+BC ranked second, with values about 9.5% and 10.5% lower than CK, respectively. CF+COF and CF showed lower levels, decreasing by about 25.1% and 30.4% relative to CK. At the R4 and R6 stages, CF+BC had the highest available P, 30.7% and 17.6% higher than CK, respectively; CF+COF ranked second, with an increase of about 24.0% over CK at R4.

In the soybean season, available P varied among treatments across sampling stages. At R2, CF+BC showed the highest available P, about 19.8% higher than CK; CF+COF ranked second with an increase of about 19.1%; CF+HA increased available P by about 16.1%; and CF by about 4.9%. At R6, CF+COF further increased available P, about 34.7% higher than CK; CF+HA increased it by about 21.9%; CF+BC by about 3.9%; and CF was about 9.2% lower than CK. At R8, CF+BC again showed the highest available P content, about 41.1% higher than CK, followed by CF+COF with an increase of about 32.6%.

#### 2.2.4. Effects of Different Fertilization Treatments on Available Potassium

**Figure 5:**
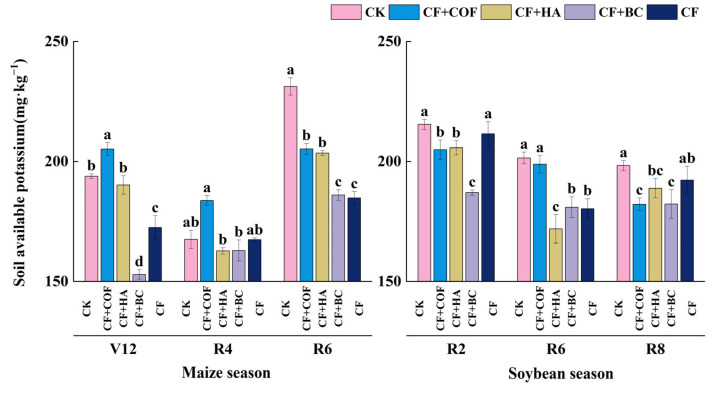
Effects of combined application of different organic materials and mineral fertilizer on available potassium content in meadow albic soil. Note: Identical letters within the same group indicate no statistically significant difference (*p* > 0.05), while different letters indicate significant differences (*p* < 0.05). Treatments: CK, no fertilizer; CF, mineral fertilizer; CF+COF, mineral fertilizer + carbon-based organic fertilizer; CF+HA, mineral fertilizer + humic acid organic fertilizer; CF+BC, mineral fertilizer + biochar-based fertilizer.

Figure 5 shows clear treatment-dependent differences in available K across crop stages. In general, CF+COF was associated with relatively higher available K in the early maize season, whereas CF+BC tended to show larger reductions across stages.

At the V12 and R4 stages of the maize season, CF+COF had the highest available K content, being 5.9% and 9.7% higher than CK, respectively. At R4, CF+HA and CF+BC both showed available K levels slightly lower than CK. By R6, CK showed the highest available K, and all fertilized treatments were lower than CK, with larger reductions under CF+BC and CF.

In the soybean season, available K also varied among treatments across sampling stages. CF+BC generally showed the lowest available K at R2, whereas CF+HA had the lowest value at R6. At R8, CK maintained the highest available K content, while all fertilized treatments remained below CK.

#### 2.2.5. Effects of Different Fertilization Treatments on Organic Carbon

**Figure 6:**
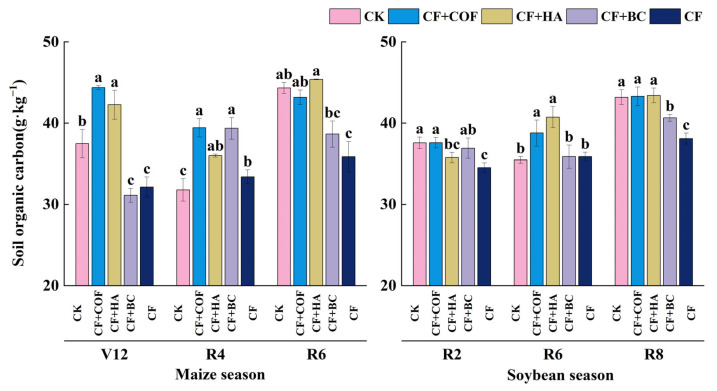
Effects of combined application of different organic materials and mineral fertilizer on organic carbon content in meadow albic soil. Note: Identical letters within the same group indicate no statistically significant difference (*p* > 0.05), while different letters indicate significant differences (*p* < 0.05). Treatments: CK, no fertilizer; CF, mineral fertilizer; CF+COF, mineral fertilizer + carbon-based organic fertilizer; CF+HA, mineral fertilizer + humic acid organic fertilizer; CF+BC, mineral fertilizer + biochar-based fertilizer.

As shown in Figure 6, overall, CF+COF significantly increased soil organic C in the early maize season; CF+HA produced larger increases in the late maize season and mid-soybean season; and CF+BC caused relatively small overall changes.

In the maize season at V12, CF+COF had the highest organic C content, 18.4% higher than CK; CF+HA ranked second, 12.8% higher than CK. In contrast, CF+BC and CF were 16.9% and 14.2% lower than CK, respectively. At R4, CF+COF and CF+BC showed the highest organic C levels, 24.0% and 23.8% higher than CK; CF+HA and CF were 13.3% and 5.1% higher than CK, respectively. At R6, CF+HA had the highest value, 2.3% higher than CK, whereas CF+BC and CF were 12.8% and 19.1% lower than CK.

In the soybean season, organic C differed among treatments across sampling stages. At R2, CF showed the lowest organic C, 8.1% below CK; CF+HA and CF+BC were 4.8% and 1.7% lower than CK, respectively. At R6, CF+HA reached the highest level, 14.8% higher than CK, followed by CF+COF with a 9.3% increase. At R8, CF+BC was 5.9% lower than CK, and CF remained the lowest, 11.8% below CK.

#### 2.2.6. Effects of Different Fertilization Treatments on Total Nitrogen

**Figure 7:**
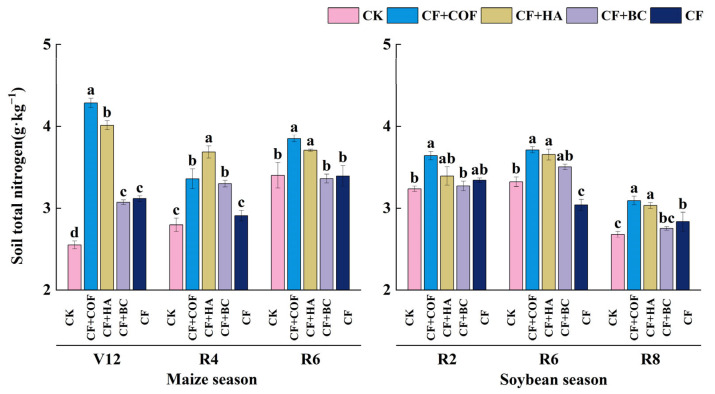
Effects of combined application of different organic materials and mineral fertilizer on total nitrogen content in meadow albic soil. Note: Identical letters within the same group indicate no statistically significant difference (*p* > 0.05), while different letters indicate significant differences (*p* < 0.05). Treatments: CK, no fertilizer; CF, mineral fertilizer; CF+COF, mineral fertilizer + carbon-based organic fertilizer; CF+HA, mineral fertilizer + humic acid organic fertilizer; CF+BC, mineral fertilizer + biochar-based fertilizer.

As shown in Figure 7, overall, CF+COF produced the most pronounced increase in total N in the early maize season; CF+HA also clearly enhanced total N in the mid–late maize season and mid-soybean season; and CF+BC led to relatively limited increases.

In the maize season at V12, CF+COF had the highest total N content, 68.0% higher than CK, followed by CF+HA with a 57.3% increase; CF+BC and CF were 20.4% and 22.1% higher than CK, respectively. At R4, CF+HA showed the highest total N, 31.8% above CK; CF+COF and CF+BC ranked second, 20.2% and 18.0% higher than CK, while CF was 4.0% higher than CK. At R6, CF+COF and CF+HA remained significantly higher than CK, by 13.2% and 9.0%, respectively, whereas CF+BC and CF showed no significant differences from CK.

In the soybean season, total N showed stage-dependent variation among treatments. At R2, CF+COF showed the highest total N, 12.6% higher than CK; CF+HA was 4.9% higher; CF+BC was 1.1% lower than CK; and CF was 3.3% higher than CK. At R6, CF+COF and CF+HA remained the highest, 11.8% and 10.1% above CK, respectively; CF+BC was 5.5% higher; and CF was 8.4% lower than CK. At R8, CF+COF and CF+HA again had the highest total N, 15.5% and 13.3% higher than CK; CF ranked next with a 5.8% increase, and CF+BC was 2.8% higher than CK.

### 2.3. Effects of Different Fertilization Treatments on Soil Enzyme Activities

#### 2.3.1. Effects of Different Fertilization Treatments on Sucrase Activity

**Figure 8:**
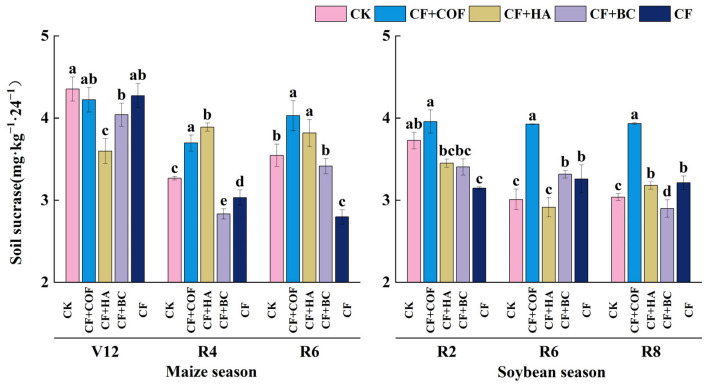
Effects of combined application of different organic materials and mineral fertilizer on sucrase activity in meadow albic soil. Note: Identical letters within the same group indicate no statistically significant difference (*p* > 0.05), while different letters indicate significant differences (*p* < 0.05). Treatments: CK, no fertilizer; CF, mineral fertilizer; CF+COF, mineral fertilizer + carbon-based organic fertilizer; CF+HA, mineral fertilizer + humic acid organic fertilizer; CF+BC, mineral fertilizer + biochar-based fertilizer.

Figure 8 indicates clear treatment-dependent variation in invertase (sucrase) activity across the two crop seasons. In general, CF+COF showed stronger enhancement during the mid–late maize season and at most soybean stages, whereas CF+HA had a more pronounced effect in the mid-maize season. By contrast, CF+BC showed relatively small fluctuations.

In the maize season at V12, CK showed the highest invertase activity, significantly higher than all fertilized treatments. CF+COF and CF ranked second, with activities about 3.0% and 1.9% lower than CK, respectively; CF+BC was about 7.2% lower than CK; and CF+HA was the lowest, about 17.4% lower than CK. At R4, CF+HA had the highest invertase activity, 19.1% higher than CK; CF+COF ranked second, 13.1% higher than CK; CF was 7.2% higher than CK; and CF+BC was the lowest, 13.3% lower than CK. At R6, CF+COF and CF+HA were both significantly higher than CK, by 13.6% and 7.7%, respectively, whereas CF showed the lowest activity, 21.1% lower than CK.

In the soybean season, invertase activity varied among treatments across sampling stages. At R2, CF+COF showed the highest activity, 6.2% higher than CK; CF was the lowest, 15.6% lower than CK; and CF+HA and CF+BC were 7.4% and 8.6% lower than CK, respectively. At R6, CF+COF again showed the highest activity, 30.5% higher than CK, followed by CF+BC (10.2% higher than CK) and CF (8.3% higher than CK), while CF+HA was the lowest, 3.3% below CK. At R8, CF+COF still had the highest invertase activity, 29.5% higher than CK; CF+HA and CF ranked next, 4.7% and 5.8% higher than CK, respectively; and CF+BC was the lowest, 4.6% lower than CK.

#### 2.3.2. Effects of Different Fertilization Treatments on Catalase Activity

**Figure 9:**
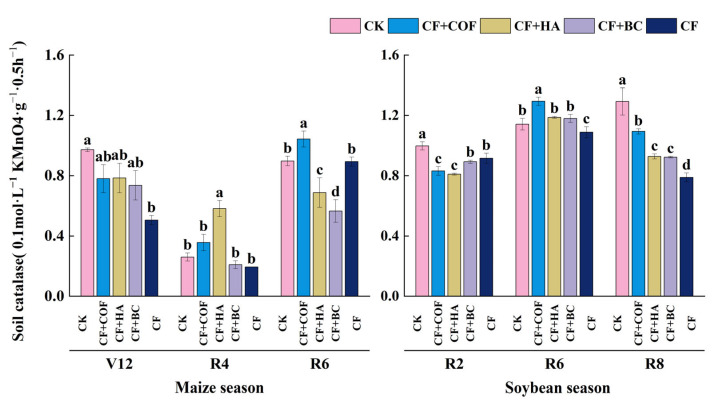
Effects of combined application of different organic materials and mineral fertilizer on catalase activity in meadow albic soil. Note: Identical letters within the same group indicate no statistically significant difference (*p* > 0.05), while different letters indicate significant differences (*p* < 0.05). Treatments: CK, no fertilizer; CF, mineral fertilizer; CF+COF, mineral fertilizer + carbon-based organic fertilizer; CF+HA, mineral fertilizer + humic acid organic fertilizer; CF+BC, mineral fertilizer + biochar-based fertilizer.

As shown in Figure 9, overall, CF+COF markedly enhanced catalase activity in the late maize season and mid-soybean season; CF+HA produced a pronounced increase in the mid-maize season; and CF+BC generally maintained relatively low activity.

In the maize season at V12, CK had the highest catalase activity. CF+COF, CF+HA, and CF+BC followed, with activities of about 19.7%, 19.2%, and 24.2% lower than CK, respectively, while CF showed the lowest activity, about 48.0% lower than CK. At R4, CF+HA showed the highest catalase activity, 123.5% higher than CK; CF+COF ranked second, 36.9% higher than CK; CF+BC and CF were 19.5% and 25.5% lower than CK, and CK itself remained at a relatively low level. At R6, CF+COF had the highest catalase activity, 16.2% higher than CK; CK ranked second; CF+HA, CF+BC, and CF were 23.4%, 37.0%, and 0.4% lower than CK, respectively.

In the soybean season, catalase activity showed clear variation among treatments across sampling stages. At R2, CK showed the highest activity; CF+COF and CF+HA were 16.6% and 18.8% lower than CK; and CF+BC and CF were 10.5% and 8.2% lower than CK, respectively. At R6, CF+COF had the highest activity, 13.3% higher than CK; CF+HA and CF+BC were 4.0% and 3.3% higher than CK; and CF was 4.7% lower than CK. At R8, CK again showed the highest catalase activity; CF+COF ranked second, 15.4% lower than CK; CF+HA and CF+BC were 28.2% and 28.6% lower than CK; and CF showed the lowest activity, 39.0% below CK.

#### 2.3.3. Effects of Different Fertilization Treatments on Urease Activity

**Figure 10:**
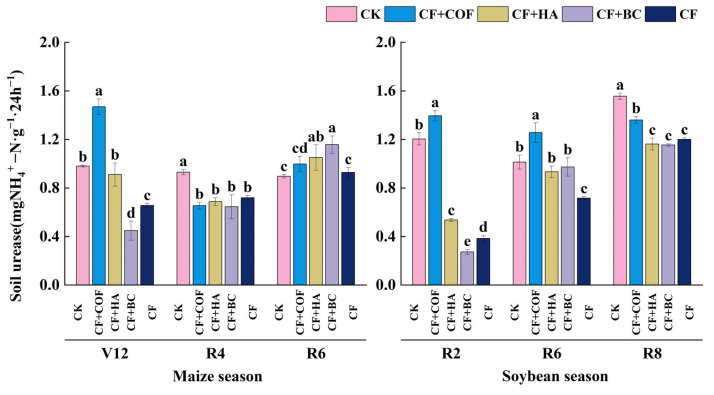
Effects of combined application of different organic materials and mineral fertilizer on urease activity in meadow albic soil. Note: Identical letters within the same group indicate no statistically significant difference (*p* > 0.05), while different letters indicate significant differences (*p* < 0.05). Treatments: CK, no fertilizer; CF, mineral fertilizer; CF+COF, mineral fertilizer + carbon-based organic fertilizer; CF+HA, mineral fertilizer + humic acid organic fertilizer; CF+BC, mineral fertilizer + biochar-based fertilizer.

Figure 10 shows that urease activity differed considerably among treatments across crop stages. Overall, CF+COF showed stronger enhancement in the early maize and early soybean stages, whereas CF+BC showed a more pronounced increase at the maize R6 stage.

In the maize season at V12, CF+COF had the highest urease activity, 49.7% higher than CK, whereas CF+BC showed the lowest value. At R4, CK maintained the highest urease activity and all fertilized treatments were lower than CK. By R6, CF+BC and CF+HA showed the highest activities, whereas CK showed the lowest value.

In the soybean season, CF+COF showed the highest urease activity at both R2 and R6, while CF remained consistently low. At R8, CK showed the highest urease activity, followed by CF+COF, whereas the other fertilized treatments remained lower than CK.

#### 2.3.4. Effects of Different Fertilization Treatments on Phosphatase Activity

**Figure 11:**
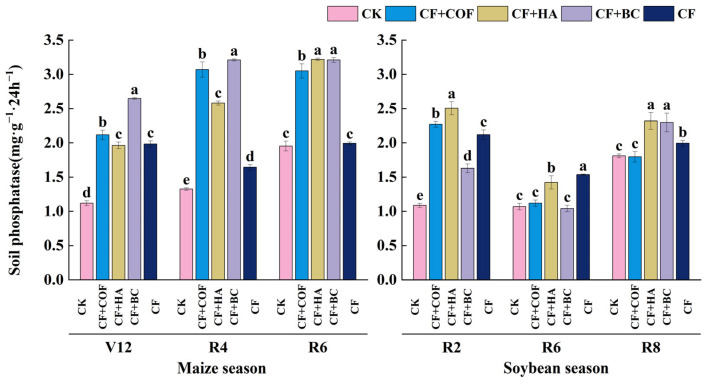
Effects of combined application of different organic materials and mineral fertilizer on phosphatase activity in meadow albic soil. Note: Identical letters within the same group indicate no statistically significant difference (*p* > 0.05), while different letters indicate significant differences (*p* < 0.05). Treatments: CK, no fertilizer; CF, mineral fertilizer; CF+COF, mineral fertilizer + carbon-based organic fertilizer; CF+HA, mineral fertilizer + humic acid organic fertilizer; CF+BC, mineral fertilizer + biochar-based fertilizer.

As shown in Figure 11, overall, CF+BC produced the most pronounced enhancement of phosphatase activity in the early maize and mid-maize season; CF+HA led to larger increases in the late maize season and early soybean season; and CF+COF also significantly elevated phosphatase activity at multiple stages.

In the maize season at V12, CF+BC had the highest phosphatase activity, 136.7% higher than CK; CF+COF ranked second, 89.2% higher than CK; CF+HA and CF were 75.3% and 77.2% higher than CK, respectively; and CK was the lowest. At R4, CF+BC again showed the highest activity, 142.2% higher than CK; CF+COF was next, 131.6% higher; CF+HA and CF were 94.7% and 23.9% higher than CK, respectively; and CK remained the lowest. At R6, CF+HA and CF+BC were both significantly higher than CK, by 65.0% and 64.5%; CF+COF and CF were 56.3% and 2.0% higher than CK, respectively, while CK was the lowest.

In the soybean season, phosphatase activity differed among treatments across sampling stages. At R2, CF+HA showed the highest activity, 130.2% higher than CK; CF+COF and CF followed, 108.8% and 94.8% higher than CK; CF+BC was 49.6% higher than CK; and CK was the lowest. At R6, CF showed the highest phosphatase activity, 43.7% higher than CK; CF+HA was next, 32.9% higher than CK; and CF+COF, CF+BC, and CK showed no significant differences. At R8, CF+HA and CF+BC had the highest activities, 28.1% and 26.9% higher than CK; CF ranked next, 10.2% higher than CK; and CF+COF did not differ significantly from CK.

### 2.4. Effects of Different Fertilization Treatments on Crop Yield and 100-Grain Weight

#### 2.4.1. Effects of Different Fertilization Treatments on Crop Yield

**Figure 12:**
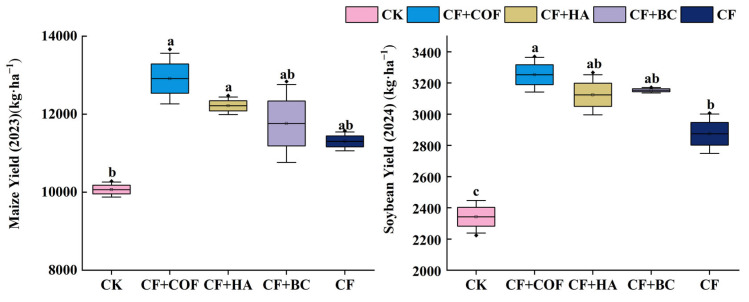
Effects of combined application of different organic materials and mineral fertilizer on crop yield in meadow albic soil. Note: Identical letters within the same group indicate no statistically significant difference (*p* > 0.05), while different letters indicate significant differences (*p* < 0.05). Treatments: CK, no fertilizer; CF, mineral fertilizer; CF+COF, mineral fertilizer + carbon-based organic fertilizer; CF+HA, mineral fertilizer + humic acid organic fertilizer; CF+BC, mineral fertilizer + biochar-based fertilizer.

Yield responses differed clearly among treatments in both crop seasons (Figure 12). Among the fertilized treatments, CF+COF produced the largest yield increase in both maize and soybean, whereas CF+HA and CF+BC also showed clear positive effects. By comparison, CF alone resulted in relatively smaller yield gains.

In the maize season, CF+COF achieved the highest yield, 28.3% higher than CK. CF+HA ranked second, with a 21.3% increase over CK; CF+BC and CF increased yield by 16.8% and 12.3%, respectively, while CK had the lowest yield.

In the soybean season, CF+COF again produced the highest yield, 38.9% higher than CK. CF+HA and CF+BC also markedly increased yield, by about 33.4% and 34.6% compared with CK, respectively; CF increased yield by 22.7%, and CK remained the lowest.

#### 2.4.2. Effects of Different Fertilization Treatments on 100-Grain Weight

**Figure 13:**
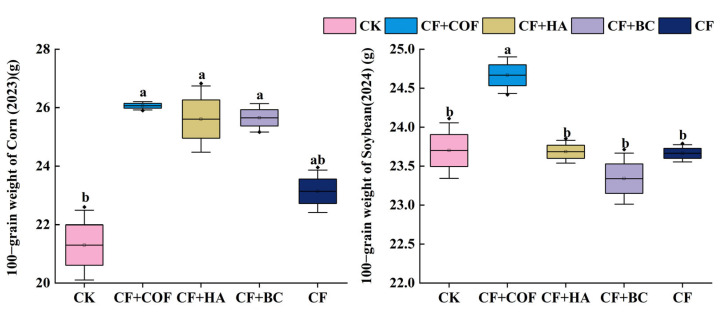
Effects of combined application of different organic materials and mineral fertilizer on 100-grain weight of crop in meadow albic soil. Note: Identical letters within the same group indicate no statistically significant difference (*p* > 0.05), while different letters indicate significant differences (*p* < 0.05). Treatments: CK, no fertilizer; CF, mineral fertilizer; CF+COF, mineral fertilizer + carbon-based organic fertilizer; CF+HA, mineral fertilizer + humic acid organic fertilizer; CF+BC, mineral fertilizer + biochar-based fertilizer.

As shown in Figure 13, overall, CF+COF significantly increased 100-grain weight in the maize season and also maintained a relatively high level in the soybean season; CF+HA and CF+BC markedly increased 100-grain weight in maize, but showed no significant differences from CK in soybean.

In the maize season, all organic-material treatments significantly increased 100-grain weight. CF+COF showed the largest improvement, 22.4% higher than CK; CF+HA and CF+BC increased 100-grain weight by 20.2% and 20.4%, respectively, with no significant difference among the three. CF increased 100-grain weight by 8.7%.

In the soybean season, CF+COF had the highest 100-grain weight, but it was only 4.1% higher than CK; CF+HA, CF+BC, and CF were essentially comparable to CK.

### 2.5. Spearman Correlation Analysis Among Soil Physicochemical Properties, Enzyme Activities, and Aggregate-Size Distribution, and Mantel Tests of Their Relationships with Yield and 100-Grain Weight

**Figure 14:**
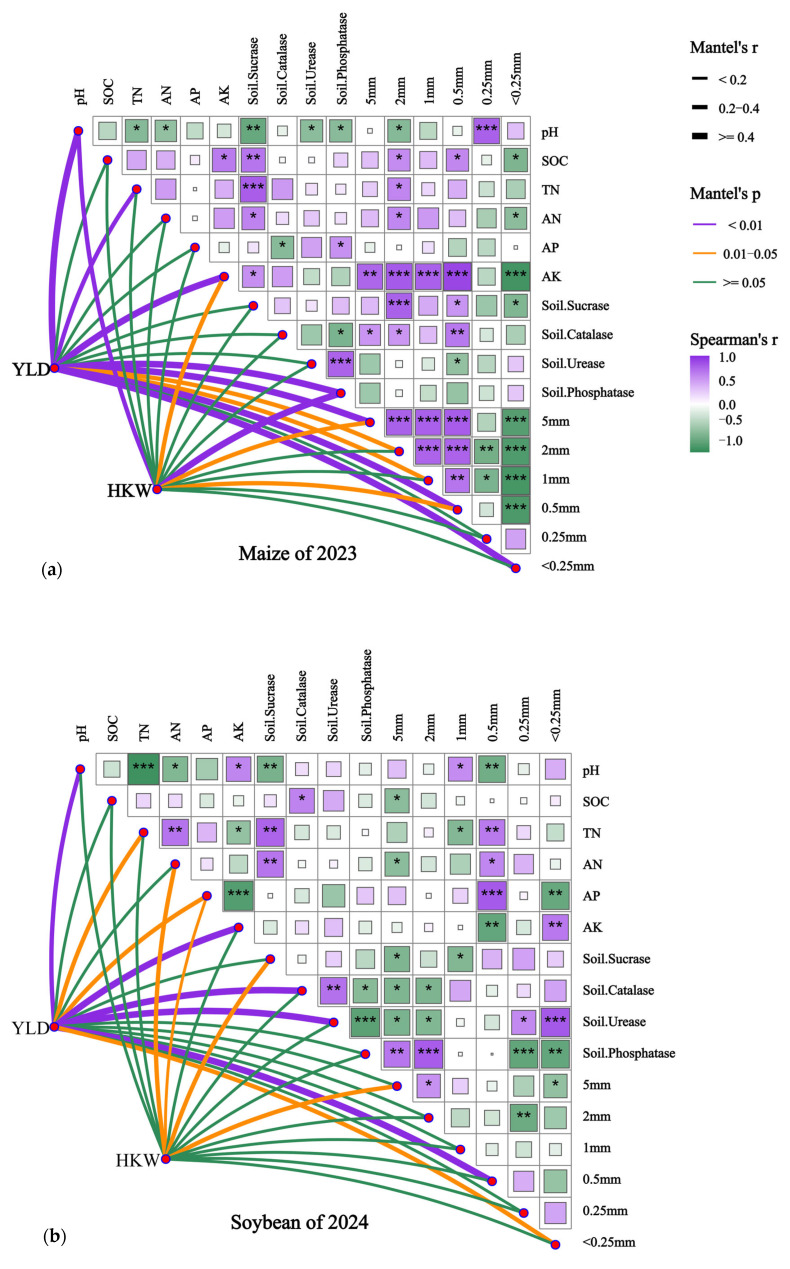
Spearman correlations among soil physicochemical properties, enzyme activities, and aggregate-size fractions, and Mantel correlations of yield and 100-grain weight with these variables in maize (2023) and soybean (2024). Note: Panels (**a**,**b**) show the results for maize in 2023 and soybean in 2024, respectively, using data from the maturity stage. Line color indicates the significance level of the Mantel test: purple, *p* < 0.01; orange, 0.01 ≤ *p* < 0.05; and green, *p* ≥ 0.05. Line thickness represents Mantel’s r value: thin, r < 0.2; medium, 0.2 ≤ r < 0.4; and thick, r ≥ 0.4. Colored squares indicate Spearman’s rank correlation coefficients, ranging from green (negative correlation) to white (no correlation) to purple (positive correlation). Asterisks indicate significant Spearman correlations: *, *p* < 0.05; **, *p* < 0.01; ***, *p* < 0.001. Abbreviations: YLD, yield; HKW, 100-grain weight; pH, soil pH; SOC, soil organic carbon; TN, total nitrogen; AN, alkali-hydrolyzable nitrogen; AP, available phosphorus; AK, available potassium. The labels 5 mm, 2 mm, 1 mm, 0.5 mm, and 0.25 mm in the figure represent aggregate fractions retained by the corresponding sieves, whereas <0.25 mm represents the fraction passing through the 0.25 mm sieve.

As shown in Figure 14, the Spearman correlation analysis and Mantel tests (*p* < 0.05) indicated that soil properties, enzyme activities, and aggregate-size distribution in both crop seasons were interrelated to varying degrees, and that the Mantel associations of yield and 100-grain weight with soil indicators exhibited clear seasonal differences. Overall, in the maize season, AK was more strongly linked with aggregate-size fractions and yield traits, whereas in the soybean season, N and P nutrients, enzyme activities, and aggregate-size fractions jointly played more prominent roles.

In the 2023 maize season, Spearman analysis showed that AK was significantly positively correlated with the larger aggregate fractions represented by the 5 mm, 2 mm, 1 mm, and 0.5 mm sieve classes, and significantly negatively correlated with the <0.25 mm fraction, indicating a close relationship between available K and increases in relatively larger aggregate fractions. SOC was significantly positively correlated with invertase and catalase activities, suggesting a close association between soil organic C accumulation and enhanced soil biochemical activity. Mantel tests further revealed that YLD was significantly associated with pH, TN, AK, and several aggregate-size fractions, with AK and larger aggregate fractions showing particularly strong relationships; HKW was significantly correlated with AK, phosphatase, and some aggregate-size fractions.

In the 2024 soybean season, Spearman analysis indicated that AP was significantly positively correlated with the aggregate fraction represented by the 0.5 mm sieve class and negatively correlated with the <0.25 mm fraction; AK showed a significant negative correlation with the 0.5 mm sieve class and a significant positive correlation with the <0.25 mm fraction, suggesting that the responses of P and K to aggregate-size distribution differed from those in the maize season. Meanwhile, TN and AN showed positive correlations with invertase activity, indicating a close coupling between N status and soil C transformation processes. Mantel tests showed that YLD was significantly associated with AK, catalase, urease, and certain aggregate-size fractions, whereas HKW was mainly and significantly related to TN, AN, AP, and some aggregate-size fractions.

## 3. Discussion

In the present study, the three organic amendment treatments differed clearly in their effects on soil aggregate composition, indicating distinct pathways of structural improvement in meadow albic soil. Changes in aggregate-size distribution are closely associated with soil aeration, water infiltration, and nutrient retention, and therefore provide an important structural basis for crop growth and yield formation. Carbon-based organic fertilizer (COF), which contains relatively labile organic components and decomposes more rapidly, may facilitate the binding of soil particles during the early stages of crop growth [27,28]. Humic acid (HA), as a natural macromolecular organic substance rich in carboxyl, phenolic hydroxyl, and other active functional groups, has strong surface activity and complexation capacity and may therefore contribute more to the integration and stabilization of existing soil structural units [29,30]. Biochar (BC), owing to its greater stability and slower nutrient-release pattern, was more often associated with higher proportions of larger structural units during the later stages of the rotation cycle. This response may be related to its high porosity, which increases adsorption sites and influences soil water and nutrient retention. In addition, the pore surfaces of biochar can provide favorable microhabitats, thereby indirectly promoting aggregate formation and the persistence of larger structural units [31,32,33]. These findings indicate that meadow albic soil amelioration does not occur through a single pathway; rather, different organic amendments may regulate structural-unit composition through different mechanisms. In meadow albic soils that are prone to plow-layer compaction, optimizing aggregate-size composition is generally beneficial for improving soil structure and may further influence nutrient retention and the root growth environment [34,35,36]. Therefore, the observed differences in aggregate composition among treatments not only reflect differential soil-improvement effects, but also suggest that their subsequent influences on nutrient availability and crop yield may be mediated through different pathways.

In the present study, the three organic amendments also differed in their effects on soil chemical properties. In this study, the three organic amendments had distinct effects on nutrient-related indicators. COF mainly increased alkali-hydrolyzable N and, at certain growth stages, maintained relatively high available K, suggesting a stronger short-term nutrient supply capacity. HA showed regulatory effects on alkali-hydrolyzable N and available P, whereas BC was particularly effective in maintaining a relatively high soil pH and increasing available P during the later growth stages. These differences suggest that the regulation of soil chemical properties by different organic amendments is not uniform, but is instead related to differences in amendment composition, chemical characteristics, and nutrient-release patterns. For COF, its advantage in alkali-hydrolyzable N may be related to the input of relatively labile organic components, which could favor microbial activity and contribute to a stronger short-term soil N supply capacity [37]. However, the specific mineralization processes were not directly measured in this study. HA contains abundant carboxyl and phenolic hydroxyl groups that can complex with Fe, Al, and Ca cations and compete with phosphate for adsorption sites on mineral surfaces, which may help explain the relatively higher available P under some stages [38,39]. However, the effects of humic substances on P sorption and P availability are complex and may vary with soil mineral composition, pH, and the characteristics of the humic materials themselves [40]. The effects of BC were mainly reflected in stabilizing and optimizing the soil chemical environment, particularly through maintaining a relatively high pH and improving P availability. This general pattern is also consistent with previous syntheses showing that biochar can influence soil conditions, nutrient dynamics, and crop performance through multiple interacting mechanisms [41]. Biochar contains alkaline ash and mineral components, and its high porosity, large specific surface area, and reactive surface sites may influence P adsorption–desorption processes, thereby contributing to improved P availability [42,43].

In the present study, treatment differences were also reflected in soil enzyme activities. Changes in enzyme activity provide insight into how different organic amendments may regulate biochemical processes in meadow albic soil. In this study, CF+COF markedly enhanced sucrase activity and, at some stages, maintained relatively high catalase activity, indicating enhanced soil biochemical activity under this treatment [44,45]. Catalase is involved in H_2_O_2_ decomposition, and increased catalase activity generally indicates enhanced soil biochemical activity. Therefore, the higher sucrase activity and, at certain stages, catalase activity under CF+COF may be associated with improved nutrient transformation and crop growth [46], although the specific turnover processes were not directly quantified in the present study. CF+BC showed a more consistent relationship between phosphatase activity and higher available P, suggesting a possible association with improved soil P availability. Previous studies have shown that biochar can improve P availability not only by regulating soil pH, surface reactive sites, and P adsorption–desorption processes, but also in association with changes in phosphatase activity, thereby jointly influencing soil P cycling [47,48,49,50]. The effects of HA on enzyme activity were generally intermediate between those of COF and BC. Previous studies have reported that HA application can increase sucrase and phosphatase activities and influence soil biochemical processes through surface activity, complexation, and the regulation of nutrient adsorption behavior [51,52].

In the present study, the combined differences in soil structure, chemical properties, and enzyme activities were ultimately reflected in crop yield and yield components. All three organic amendment treatments combined with mineral fertilizer produced higher yields than mineral fertilizer alone, although the magnitude of yield increase differed among treatments. Among them, CF+COF achieved the highest yields in both maize and soybean. When considered together with the chemical and enzymatic responses, this yield advantage may be related to relatively higher short-term nutrient availability and enhanced soil biochemical activity under this treatment [53]. These interpretations should be considered together with the differences in nutrient inputs among treatments. In the maize season, all organic amendment treatments significantly increased 100-grain weight, with CF+COF showing the greatest increase, indicating that this treatment not only increased final yield but also favored grain filling. Previous studies have shown that high maize yield is closely associated with continuous N uptake and supply, and that sufficient N availability during the late growth period is beneficial for grain filling and grain-weight formation [54,55]. In soybean, by contrast, 100-grain weight did not differ significantly among treatments, although yield increased markedly, suggesting that the yield gains were more likely attributable to increases in pod and seed number rather than to heavier individual seeds [56]. Overall, these results further demonstrate that although all three organic amendments promoted yield under maize–soybean rotation on meadow albic soil, their dominant patterns of response differed. COF was more closely associated with higher short-term nutrient availability and grain-weight improvement, whereas HA and BC were more often associated with improvements in the soil environment and aggregate-related properties. These interpretations are based on the measured indicators in this study and should not be regarded as direct evidence of specific unmeasured processes. Nevertheless, these conclusions are based on a two-year fixed-site experiment, and longer-term studies are still needed to verify the stability of these effects under different environmental and management conditions. Future studies should also evaluate the economic feasibility and greenhouse-gas implications of these amendment strategies under multi-year and multi-site conditions.

The correlation and Mantel analyses further indicated a seasonal shift in the dominant drivers associated with yield formation. In the maize season, yield traits were more closely linked with available K and larger aggregate fractions, suggesting that structural improvement and K availability were particularly important under maize growth conditions. In contrast, in the soybean season, N and P nutrients together with enzyme activities and aggregate fractions showed stronger associations with yield-related variables, indicating a more integrated influence of nutrient status and soil biochemical activity. This seasonal difference may reflect the distinct nutrient demands and physiological characteristics of maize and soybean under the rotation system. Overall, these findings support our hypothesis that different organic amendments combined with mineral fertilizer differentially affect soil properties, enzyme activities, and crop yield in meadow albic soil.

## 4. Materials and Methods

### 4.1. Experimental Site

**Figure 15:**
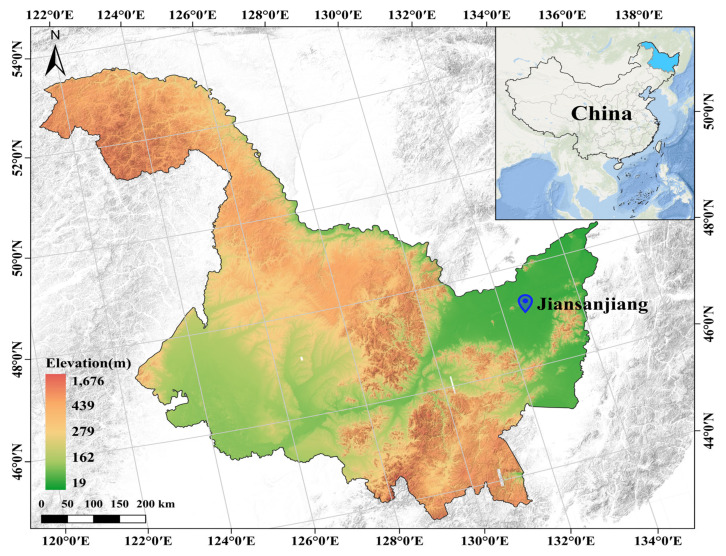
Geographical location of the experimental site.

**Figure 16:**
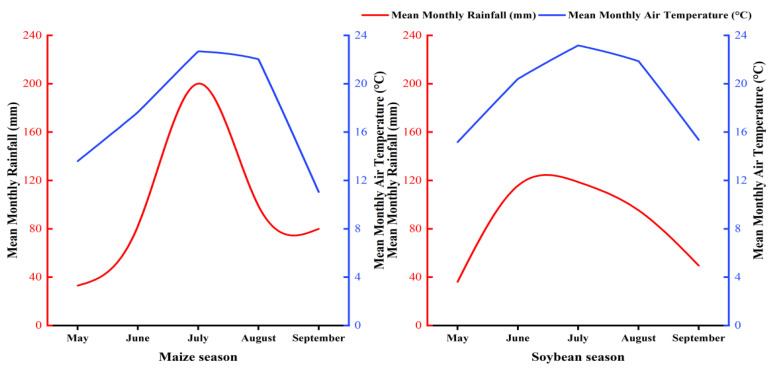
Daily mean temperature and precipitation during the growing seasons of maize (2023) and soybean (2024).

The field experiment was conducted from 2023 to 2024 at the Sixth Management Zone of Qixing Farm, Jiansanjiang City, Jiamusi, Heilongjiang Province, China (132°35′58.402″ E, 47°10′54.401″ N). The experimental site is located on an alluvial–lacustrine low plain, and the soil type is meadow albic soil (albic soil). The region has a cold temperate, humid monsoon climate, with a mean annual temperature of 3.2 °C and an average annual precipitation of 550–600 mm. The geographical location of the experimental site is shown in Figure 15. The daily mean temperature and precipitation during the crop growing seasons in 2023 and 2024 are shown in Figure 16.

### 4.2. Experimental Design

The maize cultivar used in the experiment was ‘Lihe 328′, supplied by Shanxi Lima Grant Special Grain R&D Co., Ltd. (Taiyuan, China). and the soybean cultivar was ‘Sui Nong 52′, supplied by the Suihua Branch of Heilongjiang Academy of Agricultural Sciences. (Suihua, China). In the fertilization scheme, Beifeng Hanfeng maize fertilizer (N–P_2_O_5_–K_2_O = 26–12–12) was used in the maize season of 2023, and Sinochem Fuwannong blended fertilizer (N–P_2_O_5_–K_2_O = 13–25–12) was used as the mineral fertilizer in the soybean season of 2024. The organic amendment products were purchased from Heilongjiang Hei Wo Tu Biotechnology Co., Ltd. (Qiqihar, China). The main chemical properties of each organic amendment are shown in Table 1, and the basic soil fertility status is presented in Table 2. The amendment properties listed in Table 1 were obtained from the product specifications provided by the manufacturer.

The experiment was conducted under a maize–soybean rotation system, with ridging and land preparation performed in autumn. Maize was planted in a large-ridge double-row pattern, whereas soybean was planted in a large-ridge triple-row pattern. All organic amendments were uniformly surface-applied once to the furrows after ridging and land preparation before the maize season of 2023, and were subsequently incorporated into the 0–20 cm soil layer during tillage operations. These organic amendments were not reapplied in the soybean season of 2024.

The application rates of the organic amendments were determined based on previous studies, preliminary experiments conducted by our group, product characteristics, and the soil characteristics of the study area. The quantitative differences among amendment application rates reflected differences in product formulation, nutrient concentration, and recommended agronomic use rather than an attempt to apply equal amendment mass across treatments. With reference to previous studies and the characteristics of meadow albic soil in the study region, the application rates of humic acid organic fertilizer, biochar-based fertilizer, and carbon-based organic fertilizer were set at 1500, 3000, and 6000 kg ha^−1^, respectively [57,58,59]. In the maize season, mineral fertilizer was applied using a combined basal and topdressing regime, with basal fertilizer applied before sowing and urea applied as topdressing before the jointing stage. In the soybean season, only basal fertilizer was applied, with no topdressing. No microbial inoculant or rhizobial inoculation was applied during the soybean season.

Maize was sown on 28 April 2023 and harvested on 15 September 2023; soybean was sown on 6 May 2024 and harvested on 1 October 2024. Irrigation, weed control, and pest and disease management during the whole growth period followed local standard agronomic practices.

A randomized complete block design was used, with five treatments and three replicates, giving a total of 15 plots. Each plot was 50 m in length, with a ridge width of 1.1 m and six crop rows per plot. To reduce border effects and environmental interference, one ridge of border rows was arranged on each side of the plot, and 10 m of buffer zone was reserved at both ends of the field. Thus, the actual planted length per plot was 70 m, consisting of eight ridges, with a total area of 616 m^2^. The core area for yield determination was the central six rows, i.e., 1.1 m per ridge × 6 ridges × 50 m = 330 m^2^. The row spacing was 40 cm for maize and 22 cm for soybean. The detailed fertilization scheme for each treatment is shown in Table 3, and the cumulative nutrient inputs are presented in Table 4.

### 4.3. Measurement Items and Methods

Soil sampling was conducted at the V12 (tasseling), R4 (grain filling), and R6 (physiological maturity) stages of maize in 2023, and at the R2 (full flowering), R6 (seed filling), and R8 (maturity) stages of soybean in 2024. Soil samples were collected from the 0–20 cm layer in each plot using a five-point sampling method, and the subsamples were combined into one composite sample. Part of each soil sample was air-dried and sieved for physicochemical analysis, and the remainder was used for soil enzyme assays.

#### 4.3.1. Determination of Soil Physical Properties

Soil aggregate-size distribution was determined by the wet-sieving method [60], as follows:

(1) Soil sampling and pretreatment

Sampling: In each plot, topsoil (0–20 cm) samples were collected from the central area using a five-point sampling pattern. Manure heaps, field ridges, ditches, and other atypical positions were avoided to ensure sample representativeness.

Sample pretreatment: A wooden spatula was used to remove the surface layer that might have contacted metal tools to avoid heavy-metal contamination. Subsamples from the five points were combined into a composite sample, and visible stones, plant roots, and other debris were removed. Samples were air-dried in a cool, ventilated place.

(2) Wet-sieving procedure

Sample preparation: Air-dried soil was gently broken along natural planes of weakness to preserve the original aggregate structure and avoid grinding. Approximately 500 g of soil was weighed for analysis.

Wet sieving: A nest of standard sieves with mesh sizes of 5 mm, 2 mm, 1 mm, 0.5 mm, and 0.25 mm was stacked from top to bottom. In subsequent figures and analyses, these sieve classes were used to represent the corresponding aggregate fractions. The weighed soil was placed on the top 5 mm sieve. The whole sieve set was then slowly immersed in clean water and soaked for 5–10 min to fully wet the aggregates. Afterwards, the sieves were oscillated on a wet-sieving apparatus at 30 cycles min^−1^ for 30 min. At the end of sieving, each sieve was carefully removed, and the aggregates retained on each sieve were washed into pre-weighed aluminum boxes.

Drying and weighing: The aluminum boxes were dried at 105 ± 2 °C to constant weight, cooled, and weighed. The mass of water-stable aggregates in each size class was recorded. The mass of particles < 0.25 mm was calculated by the difference between the total mass and the sum of all larger size fractions.

#### 4.3.2. Determination of Soil Chemical Properties

Soil pH was measured with a pH meter in a 1:5 soil:water suspension (*w*/*v*) [61]. Alkali-hydrolyzable N (AN) was determined by the alkaline hydrolysis diffusion method. Available P (AP) was extracted with NaHCO_3_ and determined colorimetrically using the molybdenum–antimony blue method [62]. Available K (AK) was extracted with 1 mol L^−1^ NH_4_OAc and measured by flame photometry [63]. Soil organic C (SOC) was determined by the external-heating K_2_Cr_2_O_7_ oxidation method [64]. Total N (TN) was determined by the Kjeldahl digestion method [65].

#### 4.3.3. Determination of Soil Enzyme Activities

Four soil enzyme activities were determined colorimetrically:

Invertase (sucrase) activity was measured by the 3,5-dinitrosalicylic acid (DNS) colorimetric method [66] and expressed as mg glucose g^−1^ soil 24 h^−1^ after incubation at 37 °C for 24 h.

Phosphatase activity was determined using disodium phenyl phosphate as substrate and a colorimetric method [67] with citrate buffer at pH 7.0 and expressed as mg phenol g^−1^ soil 24 h^−1^ after incubation at 37 °C for 24 h.

Urease activity was measured by the phenol-sodium hypochlorite colorimetric method [67] and expressed as mg NH_4_^+^-N g^−1^ soil 24 h^−1^ after incubation at 37 °C for 24 h.

Catalase activity was determined by titration with 0.1 mol L^−1^ KMnO_4_ [67] and expressed as mL 0.1 mol L^−1^ KMnO_4_ g^−1^ soil 0.5 h^−1^ consumed during a 30 min reaction at 25 °C.

#### 4.3.4. Determination of Crop Yield and Yield Components

For maize, in each plot, a 5 m length of one ridge (1.1 m wide) was harvested completely to record plant number and total fresh weight. In addition, 10 representative plants per plot were sampled to determine yield components, including 100-grain weight.

For soybean, 10 representative plants were collected from each plot (30 plants in total for the three replicates) and brought to the laboratory for yield component analysis, including pods per plant, seeds per plant, seed weight per plant, and 100-grain weight. After removing border rows, a 5 m length of one ridge in each plot was harvested completely, air-dried, weighed, and adjusted to a standard moisture content to calculate plot yield, which was then converted to yield per hectare.

### 4.4. Data Processing

After preprocessing the experimental data in Excel 2021 (Microsoft, Redmond, WA, USA), one-way analysis of variance (ANOVA) was performed using SPSS 26.0 (IBM, Chicago, IL, USA), and differences among treatments within each sampling stage were tested by Duncan’s multiple range test at *p* < 0.05. Figures were plotted using Origin 2024 (OriginLab, Northampton, MA, USA). Mantel tests were employed to explore the relationships between soil physicochemical indicators and crop yield traits. Differences among sampling stages were described only as temporal patterns and were not interpreted as direct statistical comparisons among phenological stages.

## 5. Conclusions

This study demonstrated that the effects of combining different organic amendments with mineral fertilizer on soil improvement and yield enhancement in a maize–soybean rotation system on meadow albic soil differed among amendment types. COF was more effective in increasing the proportion of medium-sized aggregates, enhancing alkali-hydrolyzable nitrogen content and some enzyme activities, and achieving relatively high yields in both crop seasons. HA showed comparatively balanced effects on aggregate composition and nutrient availability. BC was particularly effective in maintaining relatively high soil pH, increasing available phosphorus, and promoting the formation of larger aggregates at later growth stages. Overall, all three organic amendments combined with mineral fertilizer were beneficial for improving meadow albic soil and increasing crop yield. Under the conditions of this study, CF+COF showed the best overall performance. These findings provide useful information for selecting suitable organic amendment strategies for maize–soybean rotation systems on meadow albic soil. These results indicate that amendment-specific management strategies may be more effective than a uniform approach for improving meadow albic soil under maize–soybean rotation.

## Figures and Tables

**Table 1:** Main chemical properties of the organic amendments used in the experiment.

	Humic Acid (kg·ha^−1^)	Carbon-Based Organic Fertilizer (kg·ha^−1^)	Biochar (kg·ha^−1^)
Organic matter content (%)	30	40	45
N (%)	1.5	2.0	1.5
P_2_O_5_ (%)	1.0	2.0	2.0
K_2_O (%)	1.5	1.0	1.5
Total nutrients (%)	4.0	5.0	5.0
pH	6.0–8.0	6.5–8.5	7.5–9.5
Moisture (%)	≤30	≤30	≤25

Note: Values are provided according to the product specifications. Total nutrients represent the sum of N, P_2_O_5_, and K_2_O.

**Table 2:** Basic physicochemical properties of the soil (0–20 cm depth) before the experiment.

Year	pH	Alkali-Hydrolyzable Nitrogen (mg·kg^−1^)	Available Phosphorus (mg·kg^−1^)	Available Potassium (mg·kg^−1^)	Organic Carbon (g·kg^−1^)	Total Nitrogen (g·kg^−1^)
2023	7.42	112.69	23.52	213.91	41.84	3.36
2024	7.59	102.67	23.32	199.89	36.39	3.03

**Table 3:** Fertilization scheme for different treatments in the maize–soybean rotation field experiment (2023–2024).

Treatment	Maize 2023			Soybean 2024
	Basal Fertilizer (kg·ha^−1^)	Organic Matter Addition (kg·ha^−1^)	Topdressing (Urea) (kg·ha^−1^)	Basal Fertilizer kg·ha^−1^
CK	0	0	0	0
CF	525	0	300	300
CF+COF	525	Carbon-based organic fertilizer: 6000 kg·ha^−1^	300	300
CF+HA	525	Humic acid: 1500 kg·ha^−1^	300	300
CF+BC	525	Biochar: 3000 kg·ha^−1^	300	300

**Table 4:** Cumulative nutrient inputs under different treatments during the maize–soybean rotation field experiment (2023–2024).

Treatment	Cumulative N Input (kg ha^−1^)	Cumulative P_2_O_5_ Input (kg ha^−1^)	Cumulative K_2_O Input (kg ha^−1^)
CK	0.0	0.0	0.0
CF	313.5	138.0	99.0
CF+COF	433.5	258.0	159.0
CF+HA	336.0	153.0	121.5
CF+BC	358.5	198.0	144.0

Note: For maize in 2023, the compound fertilizer had an N–P_2_O_5_–K_2_O ratio of 26–12–12, and topdressing urea was assumed to contain 46% N. For soybean in 2024, the blended fertilizer had an N–P_2_O_5_–K_2_O ratio of 13–25–12. Organic amendments were applied only before the maize season of 2023. Nutrient inputs from the organic amendments were calculated based on the material properties shown in Table 1.

## Data Availability

The original contributions presented in this study are included in the article. Further inquiries can be directed to the corresponding author.

## Abbreviations

The following abbreviations are used in this manuscript:

CF	Mineral fertilizer
COF	Carbon-based organic fertilizer
HA	Humic acid organic fertilizer
BC	Biochar-based fertilizer
CF+COF	Mineral fertilizer combined with carbon-based organic fertilizer
CF+HA	Mineral fertilizer combined with humic acid organic fertilizer
CF+BC	Mineral fertilizer combined with biochar-based fertilizer
AN	Alkali-hydrolyzable nitrogen
AP	Available phosphorus
AK	Available potassium
SOC	Soil organic carbon
TN	Total nitrogen
YLD	Yield
HKW	100-grain weight
